# Gerosuppression in confluent cells

**DOI:** 10.18632/aging.100714

**Published:** 2014-12-31

**Authors:** Olga V. Leontieva, Mikhail V. Blagosklonny

**Affiliations:** Department of Cell Stress Biology, Roswell Park Cancer Institute, Elms and Carlson Streets, Buffalo, NY 14263, USA

**Keywords:** MTOR, rapalogs, sirolimus, aging, cancer, senescence

## Abstract

The most physiological type of cell cycle arrest – namely, contact inhibition in dense culture - is the least densely studied. Despite cell cycle arrest, confluent cells do not become senescent. We recently described that mTOR (target of rapamycin) is inactive in contact-inhibited cells. Therefore, conversion from reversible arrest to senescence (geroconversion) is suppressed. I this Perspective, we further extended the gerosuppression model. While causing senescence in regular cell density, etoposide failed to cause senescence in contact-inhibited cells. A transient reactivation of mTOR favored geroconversion in etoposide-treated confluent cells. Like p21, p16 did not cause senescence in high cell density. We discuss that suppression of geroconversion in confluent and contact-inhibited cultures mimics gerosuppression in the organism. We confirmed that levels of p-S6 were low in murine tissues in the organism compared with mouse embryonic fibroblasts in cell culture, whereas p-Akt was reciprocally high in the organism.

## Preface

When normal cells become confluent, they get arrested: a phenomenon known as contact inhibition [1-7]. Certainly, this is the most physiologically relevant type of cell cycle arrest. In the organism, cells are predominantly contact-inhibited. Yet, contact inhibition is the least studied type of cell cycle arrest. Instead, scientific attention has been attracted to two types of arrest: (a) starvation-induced arrest and (b) Cyclin Dependent Kinase-inhibitor (CDKi)-induced arrest.

As a classic example of starvation-induced arrest, serum withdrawal causes reversible quiescence in normal cells. During serum-starvation, mitogen-activated pathways become silent [8]. Cells neither grow in size nor cycle. Re-addition of serum causes cell activation and proliferation.

As an example of CDKi-induced arrest, DNA damage and telomere shortening induce p53, which in turn induces p21 and p16, inhibiting CDKs. In other cases, stresses induce both p21 and p16 [8-23]. When serum growth factors and nutrients stimulate growth, then inhibition of CDKs leads to senescence [8]. All stresses that induce senescence inhibit CDKs in part by inducing CDKi such as p21, p16, p15. Oncogenic Ras and Raf activate MAPK and mTOR pathways and induce p21 and p16, causing senescence [9, 24-27].

Numerous studies have been aimed to pinpoint the difference between quiescence and senescence based on either the point of arrest, the nature of stresses or peculiarities of CDKi (p21 versus p16). Yet, despite all efforts, the distinction remained elusive.

In fact, the difference between quiescence and senescence lies outside the cell cycle [8, 28, 29]. A senescent program consists of two steps: cell cycle arrest and gerogenic conversion or geroconversion, for brevity [29]. It is geroconversion that distinguishes quiescence from senescence. Geroconversion is “futile cellular growth” driven by mTOR as well as related mitogen-activated and growth-promoting signaling pathways [29-31]. Rapamycin suppresses gero-conversion, maintaining quiescence instead [32-38]. Furthermore, any condition that directly or indirectly inhibits mTOR in turn suppresses geroconversion [39-49]. Two-step model of senescence is applicable to all forms of senescence: from replicative and stress-induced to physiological cellular aging in the organism [29]. Senescent cells are hyper-active, hyper-functional (for example, hyper-secretory phenotype or SASP) compensatory signal-resistant, secondary malfunctional and eventually atrophic [28, 36-38, 50-55]. Hyper-function and secondary malfunction lead to age-related diseases from cancer and atherosclerosis to diabetes and Alzheimer's disease [54, 56-73]. MTOR-driven gero-conversion activates stem cells, eventually leading to their exhaustion [34, 46, 74-82].

Rapamycin extends life span and prevents age-related diseases, including cancer in mice and humans [33, 57-73, 83-110].

The two-step model is applicable to contact inhibition. Given that contact inhibition is reversible, we predicted that mTOR is inhibited. In fact, we found that mTORC1 targets - S6K and S6 – are dephosphorylated in CI cells [41]. Furthermore, activation of mTOR (by depletion of TSC2) shifts reversible contact inhibition towards senescence [41]. Thus, it is deactivation of mTOR that suppresses geroconversion in contact inhibited cells. Deactivation of mTOR was associated with induction of p27. In cancer cells, there is no induction of p27 in high cell density. Accordingly, cancer cells do not get arrested in confluent cultures. There is a complex relationship between p27 and mTOR [111-113].

To cause arrest of cancer cells, we induced ectopic p21. Remarkably, p21-mediated arrest, which leads to senescence of HT-p21 cells in regular density, did not cause senescence in confluent cultures [41]. Why? It turned out that the mTOR pathway was inhibited in dense cultures of cancer cells. Yet, cancer cells do not induce p27 and do not undergo contact inhibition. mTOR is constitutively activated in cancer, [114-118]. And induction of p21 by itself does not inhibit mTOR. So why mTOR is deactivated not only in contact-inhibited but also in confluent cancer cells? The answer is that cancer cells with highly increased metabolism rapidly exhaust and acidify the medium, thus inhibiting mTOR by starvation-like mechanism [41]. In fact, change of the medium restored mTOR activity. Therefore, in normal cells with low metabolism, mTOR is deactivated by contact inhibition and the change of the medium only marginally affects mTOR. In cancer cells, mTOR is inhibited due to exhaustion of the medium. And some cell lines are somewhere in between.

## Illustrations

**Figure 1:**
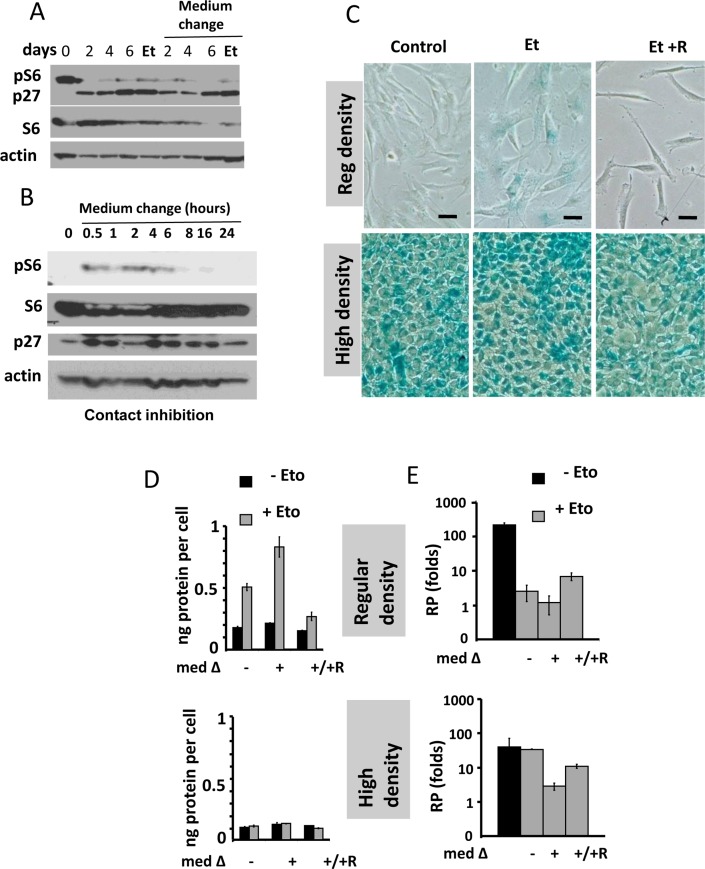
Contact inhibition suppresses etoposide-induced senescence in WI-38t cells **(A-B**) Immunoblot analysis [41]. (A) WI-38t cells [41] were plated at high density and lysed on the days indicated. When indicated “Medium change”, the medium was changed to fresh one every day. Et: cells were treated with 0.5 μg/ml etoposide on day 3 and lysed on day 6. p-S6(S240/244). (**B**) The effect of medium change on Contact Inhibited cells, measured in hours. (**C**) Beta-gal staining. WI38t cells were plated at regular or high density. After 3 days, 0.5 μg/ml etoposide (Et) and +/− 10 nM rapamycin (R) was added, if indicated. After 3 days, cells were stained for beta-Gal. Bar – 100 μm. (**D-E**) Cells were treated as described in panel C. Data are mean ± SD. (**D**) Cell size, protein per cell. (**E**) Reversibility potential or Replicative potential (RP). On day 6, cells were counted and re-plated at 1000/well in 12-well plates in fresh drug-free medium. Cells were counted after 9 days of growth. Fold increase in cell numbers were calculated. Mean ± SD.

In agreement with previous report, pS6 was barely detectable in contact inhibited cells (Fig. 1A). Inhibition of pS6 was associated with induction of p27. Treatment of contact-inhibited (CI) cells with etoposide did not affect either pS6 or p27. Change of the medium also did not affect pS6, as measured on second day after the change. Yet, the change of the medium transiently activated pS6 up to 6 hours (Fig. 1 B). This transient activation was not result in medium exhation, because CM by itseld did not inhibit pS6 in sparse culture [41]. Transient induction was in part due to hyper-sensitivity of CI-cells to slight signals.

We treated CI-culture and regular (exponentially growing) culture with etoposide for 3 days (Fig. 1C). In regular culture, WI-38 cells acquired a large flat morphology with beta-Gal staining. This senescent morphology was prevented by rapamycin (Fig. 1C). So etopiside caused mTOR-dependent senescence in regular culture conditions.

In CI-culture, cells were beta-Gal positive to start with. In fact, beta-Gal-staining is a marker of both contact inhibition, senescence and serum-starvation [119-130]. In contact-inhibition, beta-Gal staining, a marker of lysosomal overactivation, is mTOR-independent [41] and Figure 1 C. We next, employed two non-morphological tests to evaluate senescence: (a) cellular hypertrophy, measured as protein per cell and (b) the reversibility potential (RP) or the potential to restart proliferation and to regenerate cell culture after splitting in drug-free media. (Note: the reversibility also means that regenerated culture consists of cells identical to initial regular culture).

In regular culture, etoposide induced hypertrophy, which was prevented by rapamycin (Fig. 1D). In CI-culture, the cells were small and etoposide failed to cause any increase in cellular size. A small cell morphology is due to deactivated mTOR pathway in CI-culture. As expected, rapamycin did not decrease cell size further. Thus, etoposide caused mTOR-dependent hypertrophy only in regular but not in CI-culture.

The regenerative/reversibility potential can be tested after washing etoposide out. It is importantly that etoposide is easily washable [131].

In regular culture, etoposide dramatically eliminated the reversibility potential (RP), meaning that etoposide-treated cells did not proliferate after re-plating in low density in drug-free culture. This effect was in part mTOR dependent, because co-addition of rapamycin and etoposide caused a lesser loss of RP (Fig. 1E).

**Figure 2:**
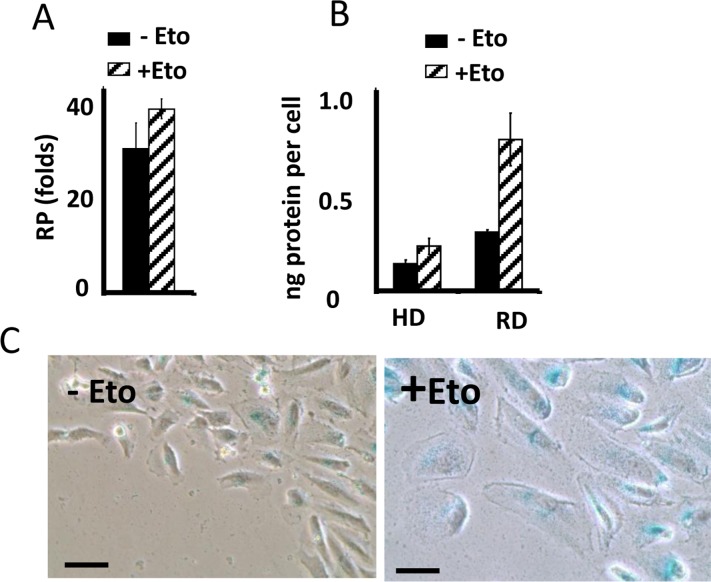
Suppression of etoposide-induced senescence in RPE cells (**A**) Reversibility potential (RP) in RPE cells [41] treated with 0.5 μg/ml etoposide (+Eto) in high cell density. After 2 day-treatment with etoposide (total 6 days in culture), cells were counted and re-plated in fresh medium at 1000/well in 24-well plates. After 7 days, cells were re-counted. Fold increase in cell numbers mean ± SD. (**B**) Protein per cell in regular (RD) versus high cell density (HD). RPE cells were plated at regular or high density. If indicated, cells were treated with 0.5 μg/ml etoposide (+Eto). After 2 days, cells were counted and lysed, protein amount was determined and protein (ng) amounts per cell was calculated. Data are mean ± SD. (**C**) Wounding. RPE cells were plated at high density. 0.5 μg/ml etoposide (+Eto) was added, if indicated “+”. Wounds were made in cell monolayer without changing the medium. After 3 days, cells were stained for beta-Gal.

In CI-culture, etoposide did not cause loss of RP. Etoposide-pretreated cells resumed proliferation, similar to untreated cells. Noteworthy, when treatment with rapamycin was combined with a daily-change of the medium, which caused transient mTOR activation in CI-culture, the cells indeed lost some RP (Fig. 1 E, low panel). This loss was m-TOR-dependent, reversed by rapamycin (Fig. 1E). Therefore, we confirmed that, due to deactivation mTOR, etoposide did not cause senescence in CI-cultures. Also, etoposide did not cause hypertrophy in CI-cultures of RPE cells, while causing hypertrophy in regular density RPE cells (Fig. 2A). Etoposide-pretreated CI-cells retained RP, capable to proliferate and regenerate culture after splitting in low cell density (Fig. 2B).

Importantly, senescence could be induced in CI-cultures by wounding in the presence of rapamycin. At the edge of wounds, mTOR is reactivated [41]. When CI-monolayer was wounded, cells acquired senescent morphology in the presence of the same medium, containing etoposide (Fig. 2C). We emphasize that failure of etoposide to induce senescence in CI-cultures cannot be explained by cell cycle arrest in CI-cultures. It was extensively studied and shown that etoposide caused DNA damage in G1 phase of the cell cycle [132-134]. Furthermore its toxicity is high in non-cycling cells [132-134].

**Figure 3:**
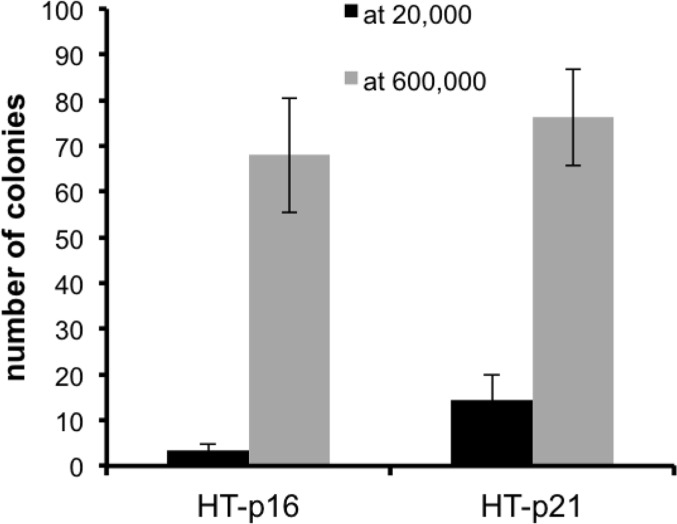
Effect of high density (HD) on p21- and p16-induced senescence HT-p21 and HT-p16 cells (see [37]) were plated at 20,000 (low density) and 600,000 (high density) cells in 6 well plates and then treated with IPTG to induce p21 and p16. After 3 days, cells were trypsinized, counted and 1000 cells were re-plated in IPTG-free medium in 6 well plates. Colonies were stained and counted after 8 days.

Previously we showed that geroconversion is suppressed in p21-arrested cancer cells in very high density [41]. This gerosuppression was associated with deactivated mTOR in exhausted media, [41]. Here we confirmed this observation and extended it to p16-induced arrest (Fig. 3). This indicated that high density suppresses geroconversion regardless of whether p21 or p16 caused arrest.

## Low basal activation of mTOR in vivo

**Figure 4:**
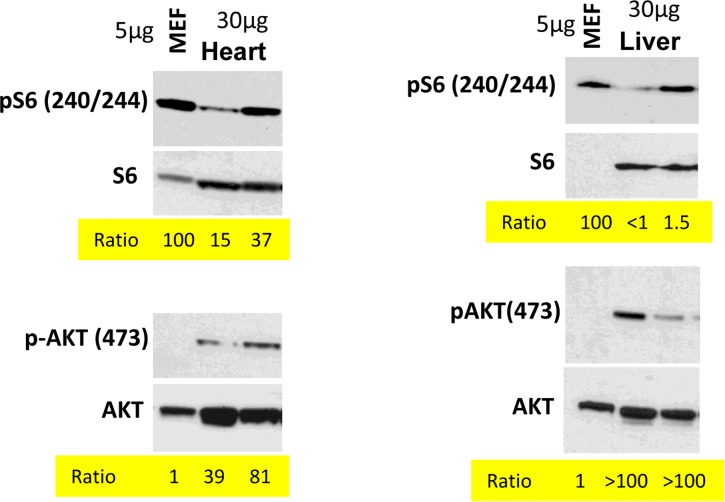
Comparison of p-S6(S240/244) and pAKT(S473) levels in murine heart and liver vs cultured MEFs Immunoblot analysis. 5 μg protein MEF lysate and 30 μg protein mice tissue were separated on the same gel and blotted for pS6/S6 and pAKT(S473)/AKT. Signal intensities were quantified using ImageJ program and normalized levels of p-S6 and p-AKT in mice organs were estimated. Ratio in MEFs is 100 and 1 (indicated as numbers). Methods are described previously and corresponding tissues samples blots published [103, 138-140].

In the organism, cells are predominantly contact inhibited. Our in vitro data predict that mTOR activity should be low in the organism compared with cells in vitro. We compared levels of p-S6 (a marker of mTOR activity) in the organs (the heart and the liver) with the levels in mouse embrionyc fibroblasts (MEF). Use of the same species (mouse) ensures that there is no species-dependent differences in detection of p-S6 by antibody. Due to extra-cell matrix in vivo, we loaded a lesser amount of MEF protein (5 microg) than tissue-extracted protein (30 microM). What was important is a ratio between pS6 and S6 and between p-Akt and Akt (Fig. 4). In the livers and the hearts, pS6/S6 ratios were lower (approximately 4-100 fold) compared to MEF.

In contrast, the ratio p-Akt/Akt was much higher in the tissues than in MEF in culture (Fig. 4).

## Physiological and clinical applications

Contact inhibition suppresses geroconversion. In the organism, most cells are contact inhibited. Even proliferating cellular pools exist in a relatively high cell density, albeit they may occupy special niches. Also, near-anoxia suppresses mTOR, thus exerting gerosuppression during cell cycle arrest [40, 130, 135, 136]. Low glucose and amino-acid levels in the organism (compared with cell culture in vitro culture conditions [137]) are also gerosuppressive. Therefore, normal cells senesce slowly in the organism. In vivo, physiological geroconversion may take decades, culminating in age-related diseases.

The gerosuppression model shed light on the treatment with DNA damaging agents. Despite DNA damage, if the organism survives, it does not become old. One of explanations is that contact inhibition is gero-suppressive. This further supports the notion that accumulation of DNA damage is not a cause of aging, which instead is driven by the mTOR pathway.

Also, the model is applicable to tumors. In tumors, necrotic regions coincide with exhaustion of the medium. Thus in large tumors (and any detectable tumors are already large), geroconversion is suppressed. This may explain lack of senescence by conventional drugs, which easily cause senescence in cell culture. Also, solitary cancer cells, trapped among contact-inhibited normal cells, such as epithelial cells are resistant to therapy-induced senescence [41]. This is a subject of our ongoing investigation.
